# New Treatment Strategies Making an Impact in Multiple Myeloma

**DOI:** 10.6004/jadpro.2017.8.3.12

**Published:** 2017-04-01

**Authors:** Charise Gleason, Jonathan Kaufman

**Affiliations:** Winship Cancer Institute of Emory University, Atlanta, Georgia

## Abstract

Even though multiple myeloma remains incurable, mean overall survival has improved dramatically as newer game-changing therapies enter the scene. At the same time, treatment decisions and the management of toxicities related to newer drug regimens are becoming more complex.

New drugs with novel mechanisms of action are making a huge impact in the treatment of multiple myeloma. At JADPRO Live 2016, these agents, their unique toxicities, and their optimal use in treating patients with newly diagnosed and relapsed myeloma were described by Charise Gleason, MSN, NP-BC, AOCNP®, and Jonathan Kaufman, MD, both of Winship Cancer Institute of Emory University, Atlanta.

Patients are living longer with multiple myeloma, but it is not yet considered curable. Although an individual’s disease course is variable, as patients with high-risk features have worse outcomes, "according to current data and trends, we can assume that a patient diagnosed in 2016 will now have a median overall survival on the order of 6 to 8 years," Dr. Kaufman predicted.

Outcomes first improved in the mid-1990s, with the development of high-dose therapy and autologous transplantation. Then came the approval of thalidomide (Thalomid), lenalidomide (Revlimid), and bortezomib (Velcade), which further changed outcomes. Then came the new immunomodulatory drug (IMiD) pomalidomide (Pomalyst) and the new proteasome inhibitor carfilzomib (Kyprolis). And in the past 4 years alone, the US Food and Drug Administration (FDA) has approved the first histone deacetylase (HDAC) inhibitor panobinostat (Farydak), the first oral proteasome inhibitor ixazomib (Ninlaro), and two monoclonal antibodies, elotuzumab (Empliciti) and daratumumab (Darzalex). Other antibodies and new classes of drugs are in development, and immunotherapies will also become part of the treatment landscape (Figure 1).

**Figure 1:**
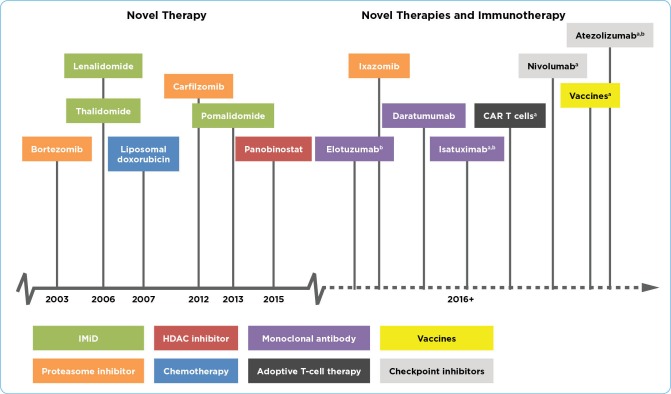
Myeloma drug development. IMiDs and proteasome inhibitors have been the backbone of treatment for some time. IMiD = immunomodulatory drug; HDAC = histone deacetylase. ^a^Not yet approved by the FDA for multiple myeloma; only available in clinical trials. ^b^Treatments studied in Multiple Myeloma Research Foundation (MMRC) trials. Image courtesy of MMRC.

## NEW CLASSIFICATIONS OF PATIENTS

"The first time we see patients, we make decisions about how to classify them," Dr. Kaufman said. The initial percentage of plasma cells allows the provider to distinguish between MGUS (monoclonal gammopathy of undetermined significance) and multiple myeloma. With MGUS, clonal protein is present but is < 10%, and the serum M protein spike is < 3 g/dL. With myeloma, clonal protein is ≥ 10%, and the M protein spike is ≥ 3 g/dL. The disease is then further subdivided into symptomatic myeloma and asymptomatic, or "smoldering," myeloma.

The current standard of care is not to initiate treatment for MGUS or smoldering myeloma (with some exceptions), as the risk of disease progression is only about 10% per year. Treatment becomes warranted when patients have symptomatic myeloma; this is often defined by and referred to as CRAB (hypercalcemia, renal impairment, anemia, bone lesions). Need for treatment may also be signaled by a myeloma-defining event: clonal plasma cells in marrow ≥ 60%, involved:uninvolved serum free light chain ratio > 100, or at least 1 focal lesion on magnetic resonance imaging. These last three signs are new to the diagnostic criteria for symptomatic myeloma, as their presence indicates a very high chance of becoming symptomatic within 6 months. "Hence, we now just initiate therapy early for these patients," he said.

The practice of withholding treatment for smoldering myeloma could change, should the Eastern Collaborative Oncology Group (ECOG) E3A06 trial show a benefit for early treatment. Based on a small Spanish study that demonstrated some advantage to treating smoldering myeloma with lenalidomide/dexamethasone (Mateos et al., 2013), a number of U.S. trials are now asking the question of whether earlier treatment will impact long-term outcomes.

The International Staging System (ISS) has also been revised (R-ISS) to include underlying biology, that is, high-risk features such as del(17p), t(4;14), and 4(14;16), and markers of proliferation, that is, lactate dehydrogenase. Five-year survival is 82% for R-ISS I patients but only 40% for R-ISS III patients (Palumbo et al., 2015). Patients can be risk-stratified into standard-risk and high-risk categories to help guide decision-making, Dr. Kaufman said.

## INDUCTION THERAPY: AGENTS, TRANSPLANT, MAINTENANCE

Eligibility for transplant drives the initial treatment decision for newly diagnosed patients. Outside of the United States, patients ≥ age 65 are usually excluded from transplant, but decisions in the United States are based as much on performance status and comorbidities, Ms. Gleason indicated.

Transplant candidates undergo induction therapy, stem cell harvesting, and autologous stem cell transplant and then receive consolidation therapy with or without maintenance. Nontransplant patients undergo induction, which is usually followed by maintenance or extended therapy.

Although the standard induction regimen for years was lenalidomide/dexamethasone, numerous trials have shown that three drugs are superior to two, and today, bortezomib is typically added to this regimen (VRd). SWOG S0777 demonstrated a 29% reduction in disease progression (*p* = .0018) and a 29% reduction in mortality (*p* = .025) with VRd vs. lenalidomide/dexamethasone alone (Durie et al., 2015). However, emphasized Ms. Gleason, VRd does increase adverse events (neurologic, pain, sensory, gastrointestinal), "so you do have to watch these patients closely."

With the availability of better drugs, can transplant be avoided? The relative benefit of early vs. delayed transplant is the subject of a phase III collaborative trial between French investigators and the Dana-Farber Cancer Institute (IMF/DFCI-2009). The control arm receives three cycles of VRd, then stem cell transplant, followed by maintenance lenalidomide/dexamethasone, with transplant performed upon relapse. The experimental arm receives VRd for 8 cycles and then maintenance lenalidomide/dexamethasone. In the French cohort, upfront transplant significantly prolonged progression-free survival, from 34 months with delayed transplant to 43 months with early transplant (*p* < .001; Attal et al., 2015). The U.S. data are not mature.

"The data point to upfront transplant as better. We think transplant will be a mainstay of treatment for some time," stated Ms. Gleason.

She added that maintenance therapy is also critical to outcomes, as it appears to extend the time to disease progression from 3 years or less to 4 years or more. "We even offer it to patients who achieve complete remissions," she noted.

## TREATMENT OF RELAPSED DISEASE

There are numerous treatment options for patients whose disease progresses. Patients have relapsed disease if, after a period of being off treatment, they require salvage therapy. Their disease is refractory if it is nonresponsive to treatment or it progresses within 60 days of the last therapy. Relapsed and refractory myeloma occurs when patients never achieve a minor response or better and become nonresponsive to salvage therapy or their disease progresses within 60 days of the last treatment.

Treatment goals are different for the different settings. With induction therapy in newly diagnosed patients, the goal is "to drive patients into remission," with no minimal residual disease. With multiple relapses and treatments, patients’ responses become shorter, and the goal is to stabilize the disease.

In choosing treatment for relapsed/refractory myeloma, providers should consider these questions: How deep and long-lasting was the patient’s prior response? How aggressive is the patient’s disease? What are the patient’s performance status and preexisting comorbidities? Are there preexisting toxicities from their disease or previous regimens? Are patients eligible for another transplant or a clinical trial?

**Figure 2:**
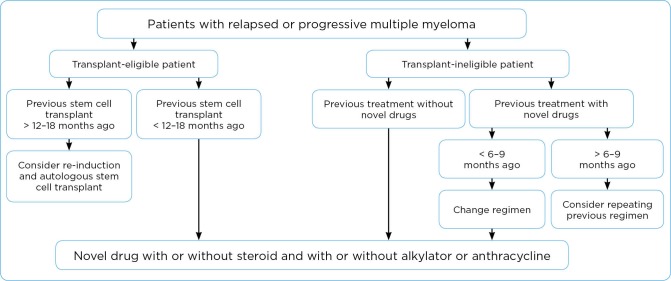
Common approach to treatment of relapsed/progressive multiple myeloma. Available clinical trial applicable to all patients. Information from Röllig et al. (2015).

Patients may benefit from another transplant, the same drugs reinitiated, or new agents (Figure 2). Newly approved agents for relapsed/refractory disease include two proteasome inhibitors—carfilzomib and ixazomib—the IMiD pomalidomide, and the HDAC inhibitor panobinostat.

## TOXICITIES OF NEW IMIDS AND PROTEASOME INHIBITORS

The speakers described the main toxicities that have been observed with newer agents and how they are dealt with in their clinic.

Pomalidomide can be myelosuppressive, so neutrophil counts must be closely monitored. Prophylaxis for deep-vein thrombosis is also required, as with any IMiD. Fatigue and constipation can be seen, but unlike some other IMiDs, peripheral neuropathy may not be a problem.

Carfilzomib also causes less neuropathy than bortezomib, but dose adjustments may be warranted for other toxicities. Cardiopulmonary toxicity can be seen, so patients with existing heart failure must be closely watched and shortness of breath assessed. Patients should be well hydrated during infusion, but clinicians should beware of overhydration. Fevers are also unique to carfilzomib, and anemia can occur in almost half the patients.

The starting dose of carfilzomib is 20 mg/m², escalated to 27 mg/m², but since neuropathy is uncommon, patients can often tolerate up to 72 mg/m². "In practice, we often give it weekly, if patients can tolerate it," Ms. Gleason said.

The use of carfilzomib is based on the ASPIRE trial, in which the addition of carfilzomib to lenalidomide/dexamethasone increased median progression-free survival by 9 months and led to deeper and longer responses (Stewart et al., 2015).

Ixazomib, which is approved in combination with lenalidomide/dexamethasone, offers the option of an all-oral regimen. In the TOURMALINE trial, ixazomib plus lenalidomide/dexamethasone reduced the risk of disease progression by 40% and overcame high-risk cytogenetic features (Moreau et al., 2015). The starting dose is 4 mg, given once a week (3 mg for moderate hepatic or severe renal impairment). Patients should take ixazomib once a week at the same time every day, 1 hour before food or 2 hours after it, and not with the dexamethasone. Antiviral prophylaxis is required, as is thromboprophylaxis when it is used with lenalidomide.

Dr. Kaufman has also prescribed ixazomib plus lenalidomide/dexamethasone as maintenance following transplant, especially in high-risk patients. Ms. Gleason added, "When we commit patients to maintenance, we now have an oral option, at least when we can get it approved for insurance."

Key adverse events include diarrhea, constipation, thrombocytopenia, peripheral neuropathy, nausea/vomiting, back pain, and peripheral edema. Flu-like symptoms may occur after the first dose, which can lead to nonadherence. "But most patients can tolerate ixazomib after a few doses, and you have the option of reducing it to 3 mg," she said.

Panobinostat, also an oral drug, is approved in combination with bortezomib/dexamethasone; it does not have single-agent activity. In the PANORAMA-1 trial, panobinostat plus bortezomib/dexamethasone led to an 83% progression-free survival and nearly doubled the complete response rate (San-Miguel et al., 2014). Dr. Kaufman and Ms. Gleason also use panobinostat with carfilzomib/dexamethasone.

Panobinostat is started at 20 mg, given every other day, for 2 consecutive weeks. Diarrhea is seen in 68% of patients and is severe in 25%. Gastrointestinal tolerability is improved with every-other-week dosing. "Ensure that antidiarrheals are at home, and avoid vitamin C and grapefruit, star fruit, and pomegranates," she advised.

Other toxicities include fatigue, nausea and vomiting, peripheral edema, low appetite, fever, myelosuppression, and electrolyte abnormalities. Patients need a baseline electrocardiogram and regular monitoring, electrolyte measurements before and after treatment, liver function monitoring, and blood cell counts.

## TOXICITIES OF NEW MONOCLONAL ANTIBODIES

"The development of monoclonal antibodies, which work via multiple mechanisms, is exciting. Two are approved, and many more are being tested," Dr. Kaufman said.

Daratumumab targets CD38 and directly induces apoptosis. Elotuzumab targets SLAMF7, a cell-surface protein on myeloma and natural killer cells. Both work by enhancing the immune system; therefore, they are unlikely to be impacted by standard resistance mechanisms. Because of their relative safety, monoclonal antibodies are being evaluated in virtually all myeloma settings, he added.

In the ELOQUENT-2 trial, elotuzumab plus lenalidomide/dexamethasone yielded a median progression-free survival of 19 months, vs. 15 months for lenalidomide/dexamethasone alone (Lonial et al., 2015). "We have patients from the original phase I study who have tolerated this regimen for more than 5 years and are doing well," Dr. Kaufman said. "We are now studying elotuzumab with other IMiDs and combinations and moving the regimen upfront, too."

More striking still are the results achieved with daratumumab, which is active as a single agent and in combination with lenalidomide/dexamethasone, he offered. "That is the one drug in myeloma that’s been associated with the most excitement. While it won’t be the final answer in management, it’s made an enormous change in how we treat," according to Dr. Kaufman.

More than one-quarter of highly refractory patients responded to single-agent daratumumab in a phase II study (Lonial et al., 2016). In combination with lenalidomide/dexamethasone, daratumumab produced clinical benefit in 88% of patients, complete responses in 34%, very good partial responses or better in 63%, and a "dramatic" progression-free survival benefit (72% at 18 months; Plesner et al., 2015).

"While daratumumab is currently approved as a single agent, the data are clear that it is very beneficial in combination," Dr. Kaufman commented. "And there is something about how it impacts the immune system that persists after we stop giving it."

The key toxicity of the monoclonal antibodies is infusion-related reaction, which is more common with daratumumab (37%) than elotuzumab (5%). Most are mild and occur in the first cycle.

Common to daratumumab are sinus/nasal congestion, throat irritation, cough, dyspnea, and wheezing. More rarely, patients can have anaphylaxis, severe hypertension, and arrhythmia. It is critical to recognize infusion reactions promptly and stop the infusion; patients can receive additional premedication and can be restarted on the antibodies at half the rate.

"We add leukotriene inhibitors to our premedications at least an hour before infusion," Dr. Kaufman said. "We have found that with the first dose, this can minimize serious reactions."

When daratumumab is used with pomalidomide/dexamethasone, myelosuppression is not uncommon. Providers should monitor blood cell counts carefully and be prepared to give growth factors, supportive care, and prophylactic antibiotics to get patients through the neutropenic period, he said.
